# IMPACT OF WORK AND ENVIRONMENTAL FACTORS ON QUALITY OF LIFE IN COMMUNITY-DWELLING SPINAL CORD INJURY INDIVIDUALS IN SOUTH KOREA USING LATENT PROFILE ANALYSIS

**DOI:** 10.2340/jrm.v57.41903

**Published:** 2025-08-20

**Authors:** Bum-Suk LEE, Boram LEE, Jincheol LIM, Onyoo KIM

**Affiliations:** 1 Department of Rehabilitation Medicine, International St. Mary’s Hospital, Incheon; 2 Division of Health Services Development for Persons with Disabilities, National Rehabilitation Center, Seoul; 3 School of Liberal Studies, Sejong University, Seoul; 4 Department of Spinal Cord Injury Rehabilitation Medicine, National Rehabilitation Center, Seoul, Korea

**Keywords:** disabled persons, quality of life, rehabilitation, spinal cord injuries

## Abstract

**Objective:**

To classify Koreans with spinal cord injury into groups with similar levels of life satisfaction and analyse the differences in life satisfaction between the groups, and identify the factors influencing their quality of life.

**Design:**

A cross-sectional study was conducted.

**Subjects:**

The International Spinal Cord Injury Survey of 711 persons with traumatic or non-traumatic spinal cord injury was used.

**Methods:**

The latent profiles were classified according to the sub-items of quality of life, and variables influencing the latent profiles were identified. The Mplus 8.0 program was used for the main analysis. Logistic regression analysis was performed to examine how the predictors were associated with latent profile membership.

**Results:**

The factors associated with a higher likelihood of belonging to the high quality of life group included marital status; having less bowel dysfunction and muscle spasms or spasticity; receiving vocational rehabilitation services and currently engaging in paid work; and negative social attitude and problematic financial status.

**Conclusion:**

Enhancing the quality of life of individuals with spinal cord injury necessitates providing medical care for bowel dysfunction or spasticity, providing vocational rehabilitation services, enabling successful return to work, improving negative perceptions regarding people with disabilities, and implementing policies to guarantee them an income.

## LAY ABSTRACT

This study aims to categorize and analyse Koreans with spinal cord injury into groups with similar levels of quality of life, and identify the factors influencing their quality of life. The International Spinal Cord Injury Survey of 711 individuals with traumatic and non-traumatic spinal cord injury was used. Improving the quality of life of individuals with spinal cord injury involves marital status, having less bowel dysfunction and muscle spasm or spasticity, receiving vocational rehabilitation services, currently engaging in paid work, negative social attitude, and problematic financial status.

According to the World Health Organization (WHO), quality of life (QOL) is defined as “individuals’ perceptions of their position in life in the context of the culture and value systems in which they live and in relation to their goals, expectations, standards and concerns” (1). After a spinal cord injury (SCI), there is a loss of sensory and motor function below the injury level, increasing the risk of secondary health conditions such as pain, spasticity, and autonomic dysfunction (2, 3). Moreover, people living with SCI experience restrictions in their daily activities and social participation, which negatively impacts their QOL and mental health (4–6).

In individuals with SCI, QOL is influenced by factors that are different from those of individuals without a disability depending on the physical and functional changes related to the injury. Health conditions (7) that occur after SCI, such as pain (8, 9), spasticity (10), and bladder dysfunction (6, 11), are known to be associated with QOL. Meanwhile, factors directly related to SCI, such as injury level and injury duration, have either been found to have no association with QOL or have been inconsistently linked to QOL by different studies (12, 13). Moreover, factors such as unemployment (14), environmental barriers (15), and social participation (4) are highly associated with life satisfaction after SCI; thus, active involvement from the rehabilitation therapy stage and continued follow-up after returning to the community are needed.

The International Spinal Cord Injury (InSCI) Community survey aims to comprehensively describe the lived experience of individuals with SCI at the multinational level, spanning all 6 WHO regions in 2017 (16). Of the 22 countries that participated in the InSCI study, Korea showed the lowest level of QOL among high-income countries based on GDP (17). This outcome may be closely related to economic factors, such as the low employment rate in Korea (18, 19). The global average employment rate for individuals with SCI was 38%, with Europe reporting the highest rate at 51%. In contrast, South Korea showed an employment rate of 28.4%, falling below the global average (15). In terms of health, the InSCI multinational analysis identified pain, muscle spasticity, sexual dysfunction, and bowel dysfunction as common and highly prevalent secondary health problems among individuals with SCI. Notably, South Korea exhibited the highest prevalence of both pain (89.7%) and muscle spasticity (88.2%) among the 21 countries surveyed. Furthermore, Korean participants reported an average of 10.1 co-occurring secondary health conditions per person, the highest among all countries included in the study (7).

Previous studies on Koreans with SCI had a small sample size, did not use standardized questionnaires, or focused mostly on SCI (20) or complications (21) in exploring influencing factors. As the factors that influence QOL would inevitably vary depending on national, ethnic, and social circumstances, studies on QOL for Koreans with SCI that consider their personal, social, health, occupational, and environmental factors are needed. Existing studies on identifying influence factors for QOL followed the variable-centred approach using correlation analysis, regression analysis, and structural equation methods. These studies focused on exploring the associations between variables under the assumption that the responses to these variables appear commonly in all samples (22). As individual differences exist in the QOL of patients with SCI, it is necessary to use a person-centred approach that can examine the samples’ heterogeneity. The present study used the latent profile method. Through this, subjects who share similar characteristics in QOL were classified, which allowed analysis that accounted for individual differences in the samples.

This study aimed to use latent profiles to classify Koreans with SCI into groups with similar levels of QOL and to analyse the differences in QOL between the groups. Additionally, it aimed to identify the factors influencing QOL among various sociodemographic, health conditions, job, and environmental factors.

## METHODS

### Data collection

The participants took part in the International Spinal Cord Injury Survey (InSCI) in South Korea from March to October 2017. Individuals with SCI were randomly selected following the InSCI research protocol in the National Rehabilitation Center and the Korea Spinal Cord Injury Association (16). A total of 6,355 community-dwelling SCI individuals were invited through text messages, emails, face-to-face interaction, and phone calls. Inclusion criteria were community-dwelling Korean aged ≥ 19 years with traumatic or non-traumatic SCI who were not currently hospitalized for rehabilitation and could understand the contents of the questionnaire. Exclusion criteria were individuals with congenital SCI, or with another neurodegenerative disease, or who had peripheral nerve injury. Accordingly, data were collected from 892 individuals. After excluding individuals with missing responses, a total of 711 individuals with SCI (539 males and 172 females) were included in the final analysis.

### Measures

QOL outcome values, which were selected from the InSCI survey, were assessed by a selection from WHO QOL-BREF. WHO QOL-BREF consists of 26 items, which are used to assess various aspects such as physical well-being, psychological well-being, social relationships, and environmental factors (23). Among the WHO QOL-BREF items, those corresponding to health, routine, oneself, relations, and living conditions were selected and each item was rated on a 5-point Likert scale (no problem [1] to extreme problem [5]).

In order to explore which factors influence the QOL of individuals with SCI, we considered sociodemographic factors (sex, age, marital status), health conditions (bladder dysfunction, bowel dysfunction, muscle spasm/spasticity, pain), job (vocational rehabilitation services, disability pension, having paid work or not), and environmental factors (accessibility of public places, negative social attitude, long transport time, problematic financial status, politics). Based on previous research results (6, 8–13), personal factors such as demographics and health conditions and external factors such as job and environment were included. The details of the item, scale, and source for each variable are given in Table I.

**Table I:** Questionnaire and measurement method of variables

Factor	Variable name	Questionnaire	Source
Sociodemographic	Sex	Please indicate your gender	
	Age	What day, month, and year were you born?	MDS
	Marital status	What is your current marital status?	MDS modified
Health problem	Bowel dysfunction	For the following health problems please rate how much of a problem it was for you in the last 3 months	SCI-SCS & SCQ
	Bladder dysfunction		
	Muscle spasms, spasticity		
	Pain		
Job	Vocational rehabilitation services	Did you receive vocational rehabilitation services after your spinal cord injury?	SwiSCI Community Survey,
	Disability pension	Do you currently receive a disability pension or a similar disability benefit?	MDS modified
	Paid work	Are you currently engaged in paid work?	SwiSCI Community Survey
Environmental	Accessibility of public places	About the last 4 weeks, please rate how much these environmental factors have influenced your participation in society (Questionnaires for each variable)	NEFI
	Negative social attitude		
	Long transport time		
	Problematicfinancial status		
	Politics		
QOL	Satisfaction with health	How satisfied are you with your health?	WHOQoL5, MDS
	Satisfaction with routine	How satisfied are you with your ability to perform your daily living activities?	WHOQoL5, MDS
	Satisfaction with oneself	How satisfied are you with yourself?	WHOQoL5, MDS, BREF,
	Satisfaction with relations	How satisfied are you with your personal relationships?	WHOQoL5, MDS
	Satisfaction with living conditions	How satisfied are you with your living conditions?	WHOQoL5, MDS

Scale: Sex (1 = Male, 0 = Female); Marital status (1 = Currently married, 0 = Currently single); Health problem (1 = No problem~5 = Extreme problem); Job (1 = Yes, 0 = No); Environmental factors (1 = Not applicable~4 = Made my life a lot harder); QOL (1 = Very dissatisfied~5 = Very satisfied).

MDS: Model Disability Survey; SCI-SCS: Spinal Cord Injury Secondary Health Conditions Scale; SCQ: Self-Administered Comorbidity Questionnaire; SwiSCI: Swiss Spinal Cord Injury Cohort Study; NEFI: Nottwil Environmental Factors Inventory; WHOQoL: WHOQoL BREF Quality of Life Assessment.

### Statistical analysis

Before conducting the LPA, mean, standard deviation, and satisfaction of normality were checked through descriptive statistics for the independent variables and QOL, the dependent variable of the present study. Moreover, Pearson correlation analysis was performed to identify variables associated with QOL. Following this, the study applied a 3-step latent profile analysis to classify individuals based on their QOL levels and to explore variables associated with profile membership. The specific procedures were as follows (24). In the first step, the number of latent profiles for QOL was derived without inputting covariates. To determine the number of latent profiles, the following analyses were conducted: significance of Sample-size-Adjusted BIC (SABIC); entropy for assessing the quality of classification; and Lo–Mendell–Rubin adjusted likelihood ratio test (LMR-LRT), used for direct comparison of goodness-of-fit (GoF) between models, was checked. Specifically, the Information Criteria (IC), an information-based fitness index such as SABIC, prefers models that produce high log-likelihood values using relatively few parameters. In other words, a smaller value means a model with the optimal number of latent types, but the SABIC value generally tends to decrease as the number of profiles increases (25). Next, Entropy indicates the average classification accuracy of the estimated model based on the probability of classes. It ranges from 0 to 1, and the closer it is to 1, the more accurate the classification (26). Finally, LMR-LRT uses the Approximation of the distribution for the maximum likelihood difference between the n-1 group model and the n group model. It provides a probability value for the result. If the result is significant, it means that the n group model is superior to the n-1 group model in terms of fit. In this study, we used the latent profile classified into 2 profiles, where LMR-LRT was significant, and Entropy was the highest. In the second step, the posterior distribution of latent profiles derived in the first step was used to estimate the group to which an individual patient has the highest probability of belonging. In the third step, errors that occurred during classification of latent profiles were controlled and logistic regression analysis was conducted to identify predictors of QOL profile membership. SPSS 27.0 (IBM Corp, Armonk, NY, USA)was used for descriptive statistics and correlation analysis, while Mplus 8 (https://www.statmodel.com/) was used for latent profile analysis. Moreover, logistic regression analysis was performed with the auxiliary variables designated by R3STEP command of Mplus.

## RESULTS

Sociodemographic and SCI-related factors of the participants are given in Table II.

**Table II:** Demographics and clinical features of the participants

Characteristics (*n* = 711)	M (SD) or *n* (%)
Age (years)	53.36 (11.73)
Sex	
Male	539 (75.8%)
Female	172 (24.2%)
Aetiology of injury	
Traumatic	664 (93.4%)
Nontraumatic	47 (6.6%)
NLI	
Tetraplegia	291 (40.9%)
Paraplegia	420 (59.1%)
Extent of SCI	
Complete injury	418 (58.8%)
Incomplete injury	293 (41.2%)
Duration of SCI (months)	16.30 (9.94)
Marital status	
Yes	337 (47.4%)
No	374 (52.6%)
Bowel dysfunction	3.24 (1.37)
Bladder dysfunction	3.06 (1.41)
Muscle spasms, spasticity	3.36 (1.33)
Pain	3.44 (1.32)
Vocational rehabilitation services	
Yes	193 (27.1%)
No	518 (72.9%)
Paid work	
Yes	202 (28.4%)
No	509 (71.6%)
Accessibility of public places	2.72 (0.96)
Negative social attitude	2.75 (0.85)
Long transport time	2.95 (0.95)
Problematic financial status	2.89 (0.91)
Politics	2.95 (0.86)

NLI: lLevel of injury; AISA: American Spinal Injury Association.

The results of the descriptive statistics and correlation analysis are as indicated in Table III.

**Table III:** Correlations between sub-items of QOL and sociodemographic/health condition/job/environmental factors

	1	2	3	4	5	M	SD	Skew	kurtosis
1	1					2.46	0.99	0.16	–0.90
2	0.62**	1				2.67	1.03	–0.01	–0.89
3	0.64**	0.72**	1			2.80	1.04	–0.07	–0.81
4	0.51**	0.56**	0.64**	1		3.09	0.95	–0.38	–0.31
5	0.55**	0.57**	0.69**	0.59**	1	2.86	1.02	–0.13	–0.66
6	0.02	–0.07	–0.05	–0.05	–0.07	–			
7	–0.04	–0.07	–0.03	–0.04	0.01	53.36	11.73	–0.13	–0.31
8	0.08*	0.06	0.10**	0.06	0.10**	–			
9	–0.24**	–0.20**	–0.23**	–0.18**	–0.21**	3.24	1.37	–0.18	–1.20
10	–0.17**	–0.10**	–0.11**	–0.14**	–0.11**	3.06	1.41	–0.03	–1.28
11	–0.22**	–0.30**	–0.27**	–0.22**	–0.25**	3.36	1.33	–0.33	–1.03
12	–0.24**	–0.19**	–0.17**	–0.15**	–0.12**	3.44	1.32	–0.40	–0.99
13	0.11**	0.15**	0.11**	0.06	0.06	—			
14	0.01	–0.01	0.00	–0.01	0.01	—			
15	0.17**	0.24**	0.24**	0.17**	0.16**	—			
16	–0.16**	–0.15**	–0.15**	–0.22**	–0.17**	2.72	0.96	–0.44	–0.73
17	–0.23**	–0.25**	–0.28**	–0.29**	–0.27**	2.75	0.85	–0.40	–0.37
18	–0.19**	–0.20**	–0.22**	–0.16**	–0.22**	2.95	0.95	–0.64	–0.48
19	–0.28**	–0.24**	–0.27**	–0.21**	–0.36**	2.89	0.91	–0.49	–0.53
20	–0.22**	–0.18**	–0.22**	–0.13**	–0.27**	2.95	0.86	–0.54	–0.28

1: Satisfaction with health; 2: Satisfaction with routine; 3: Satisfaction with oneself; 4: Satisfaction with relations; 5: Satisfaction with living conditions; 6: Sex; 7: Age; 8: Marital status; 9: Bowel dysfunction; 10: Bladder dysfunction; 11: Muscle spasms, spasticity; 12: Pain; 13: Vocational rehabilitation services; 14: Disability pension; 15: Paid work; 16: Accessibility of public; 17: Negative social attitude; 18: Long transport time; 19: Problematic financial status; 20: Politics.

** *p* < 0.01,

* *p* < 0.05.

The analysis results showed that the skewness and kurtosis of all variables were distributed within the range of absolute values of 2 and 7, indicating that the normality assumption was satisfied (27). The correlation analysis results showed that sex, age, and disability pension were not significantly correlated with all 5 items of QOL, whereas other variables were significantly correlated with at least 3 items of QOL.

### Determination of number of QOL profiles (sub-groups)

To derive the number of QOL profiles, the number of profiles was increased from 2 to 4. The results are presented in Table IV. The BLRT of the model with 4 profiles was statistically significant, which confirmed that the GoF of models with fewer profiles was superior. When the GoF of models with 2 and 3 profiles was compared, LMR-LRT and BLRT were not statistically significant, but the entropy value, which is used to determine how accurately the model classifies an individual into a particular group, was highest in the model with 2 profiles (entropy = 0.858). Accordingly, the model with 2 profiles was selected as the optimal model based on comprehensive consideration of statistical criteria and interpretability.

**Table IV:** Goodness-of-fit indices for different latent profile analysis QOL model

Item	2 profiles	3 profiles	4 profiles
SABIC	8828.430	8414.610	7769.360
LMR-LRT (*p*)	1317.055*** (0.000)	423.422*** (0.000)	649.124 (0.144)
Entropy	0.858	0.845	1.000
1	329 (46.3)	212 (29.8)	84 (11.8)
2	382 (53.7)	314 (44.2)	198 (27.8)
3		185 (26.0)	228 (32.1)
4			201 (28.3)

SABIC: Sample-size Adjusted BIC; LMR-LRT: Lo–Mendell–Rubin adjusted likelihood ratio test; Entropy: quality of classification.

*** *p* < 0.001,

** *p* < 0.01,

* *p* < 0.05.

### Description of profiles

To examine the characteristics of the model with 2 profiles, the level of 5 QOL items for each profile was analysed, the results of which are given in Table V. Moreover, the results from testing whether the mean differences in the QOL items between the 2 profiles are significant were examined. When QOL sub-groups 1 and sub-group 2 were compared, the results showed that the mean values of all 5 items in sub-group 1 were statistically significantly high (*p* < 0.001). In consideration of these characteristics, sub-group 1 was named “high QOL group” and sub-group 2 was named “low QOL group”.

**Table V:** Comparison of sub-item means between 2 latent profiles

Variables	Group	Mean (SD)	*t* (df)	*p*-value
Satisfaction with health	High	3.06 (0.80)	23.58*** (709)	< 0.001
	Low	1.75 (0.67)		
Satisfaction with routine	High	3.37 (0.73)	28.50*** (702.60)	< 0.001
	Low	1.85 (0.69)		
Satisfaction with oneself	High	3.53 (0.66)	31.40*** (684.50)	< 0.001
	Low	1.94 (0.69)		
Satisfaction with relations	High	3.62 (0.66)	19.40*** (604.83)	< 0.001
	Low	2.47 (0.87)		
Satisfaction with living conditions	High	3.47 (0.77)	22.54*** (709)	< 0.001
	Low	2.15 (0.78)		

Bold values indicate statistically significant results. Significance levels are denoted as follows:

*** *p* < 0.001,

** *p* < 0.01,

* *p* < 0.05.

In the high QOL group, there were 382 patients (53.7%) and the mean value of each item was within the range of 3.06–3.62. In the low QOL group, there were 329 patients (46.3%) and the mean value of each item was within the range of 1.75–2.47.

### Exploration of variables associated with latent QOL profile membership

To explore the variables associated with latent QOL profile membership, a multinomial logistic regression analysis was conducted, using the low QOL group as the reference category (Table VI).

**Table VI:** Exploratory analysis of variables associated with quality of life using logistic regression

Factor	Variable	Exp(B)	B	S.E.	*p*
Sociodemographic	Marital status	1.66**	0.51	0.19	0.009
Health problem	Bowel dysfunction	0.82*	–0.20	0.09	0.022
	Bladder dysfunction	1.13	0.12	0.09	0.170
	Muscle spasms, spasticity	0.70***	–0.36	0.08	< 0.001
	Pain	0.91	–0.09	0.08	0.264
Work	Vocational rehabilitation services (ref = No)	1.82*	0.60	0.24	0.015
	Paid work (ref = No)	1.91**	0.64	0.24	0.006
Environmental	Accessibility of public places	0.94	–0.06	0.12	0.626
	Negative social attitude	0.52***	–0.65	0.14	< 0.001
	Long transport time	1.09	0.08	0.13	0.525
	Problematic financial status	0.52***	–0.66	0.16	< 0.001
	Politics	1.34	0.29	0.16	0.072

Bold values indicate statistically significant results. Significance levels are denoted as follows:

*** *p* < 0.001,

** *p* < 0.01,

* *p* < 0.05.

Among the sociodemographic factors, marital status was found to have a significant influence, meaning that those who are currently married had a higher likelihood of belonging to the high QOL group than the low QOL group. Among the health problem factors, fewer problems with bowel dysfunction and muscle spasms or spasticity in the past 3 months were associated with a higher likelihood of belonging to the high QOL group. Among the job factors, vocational rehabilitation services and paid work were found to have a significant influence, meaning that patients who received vocational rehabilitation services after SCI or those who were currently engaging in paid work were more likely to belong to the high QOL group. Lastly, among the environmental factors, negative social attitude and problematic financial status were found to have a significant influence, meaning that a negative social attitude toward social participation by the patients in the past 4 weeks or a problematic financial situation not making life more difficult was associated with a higher likelihood of belonging to the high QOL group.

## DISCUSSION

The present study used a person-centred approach for QOL of Koreans with SCI in order to divide them into high and low QOL groups that share similar characteristics regarding QOL to account for individual differences in each sample, while ensuring between-group heterogeneity and within-group homogeneity. The effects of sociodemographic, health problem, job, and environmental factors on 2 groups with different levels of QOL were analysed.

The findings in the study showed that health problems such as bowel dysfunction and muscle spasm or spasticity occurring after SCI had an influence on QOL. Secondary health conditions such as problematic spasticity and constipation showed independent associations with QOL in multiple regression analysis (28). Health conditions cause a decrease in occupational and community participation and an increase in health-related costs, which can lead to a decline in QOL (29). A study on 299 Koreans with SCI reported that urological and bowel problems were the second most influencing factors of QOL, only after physical disability (21). Problems with bladder (71%), bowel (61%), spasm (57%), and pain (55%) are most frequently reported after SCI (30). In the present study, neurogenic bladder did not have an influence on QOL, whereas neurogenic bowel did. In the caregiver burden for Koreans with SCI, neurogenic bowel has been reported to be associated with the most difficulty, time spent, and physical injury – more so than other care activities including neurogenic bladder (31). This could be attributed to the fact that neurogenic bladder care can reduce incontinence with methods such as transurethral catheter insertion or clean intermittent catheterization, whereas bowel care may have an influence that is more challenging to ascertain. Therefore, it is necessary for individuals with SCI to understand the secondary health problems that may occur after a neurological injury and to actively treat and manage such problems.

Paid work status and financial problems had an influence on QOL and, accordingly, vocational rehabilitation services were found to be an important factor. Individuals who are currently working showed better participation and higher QOL (4, 32). Moreover, effective vocational programmes for individuals with SCI showed positive employment programme outcomes (33). Despite advances in medical technology and various efforts to improve return to work and employment among individuals with SCI, the employment rate still remains at a low level (34). Providing specific vocational rehabilitation during primary rehabilitation can help increase the employment rate (4). In Korea, training in rehabilitation hospitals for a return to work is not covered by insurance, and there are very few institutions that can provide professional training. Accordingly, there is a need for efforts to provide vocational rehabilitation education and training for returning to work during hospitalization so as to enable patients to naturally return to paid work after discharge.

Environment and environmental barriers of individuals with SCI vary by national income level, and in most countries inaccessible buildings or places and a lack of short- and long-distance transport were identified to be high environmental barriers (35). However, Korea has strict standards for guaranteeing wheelchair access to public institutions and convenience facilities based on the Enforcement Decree of the Act on the Guarantee of Convenience Promotion of Persons with Disabilities, Senior Citizens, Pregnant Women, and Nursing Mothers. Moreover, the Act on Promotion of the Transportation Convenience of Mobility Disadvantaged Persons ensures that mobility-disadvantaged persons can safely and conveniently use public transport, such as buses and subways; in addition, vehicles are equipped with wheelchair lifts to support the movement of mobility-disadvantaged persons. In Korea, transport-related factors (accessibility of public, long transport time) had no influence on QOL, unlike in other countries. However, negative social attitude and problematic financial status had an influence on QOL. Higher life satisfaction in individuals with SCI is closely associated with social reintegration (36). Therefore, individual and societal efforts are needed to improve negative perceptions of disabilities and to achieve a stable financial situation while being a member of society.

### Study strengths and limitations

As Korea does not have a national spinal cord injury registry, it is difficult to accurately determine the incidence and prevalence of SCI (37). Consequently, there are very few surveys or research on a large number of Koreans with SCI, as in the present study. The InSCI survey aims to describe and identify the factors that determine the levels of functioning, health, and well-being of individuals with SCI based on ICF (38). A strength of the present study is that it used a representative international survey to investigate Koreans with SCI through a designated sampling method.

Moreover, the present study also investigated whether factors that are known to influence QOL through previous studies are actually protective factors or risk factors of QOL in Koreans with SCI. QOL would inevitably vary depending on national, ethnic, and social circumstances. However, most previous studies on individuals with SCI were conducted in Europe or the United States. Accordingly, the significance of the present study is that it investigated QOL in a large number of Koreans with SCI with consideration of sociodemographic, health, job, and environmental factors.

Latent profile analysis used in the present study is a method based on mixture modelling, which identifies the response patterns of individuals and classifies heterogeneous sub-groups within a group that shares common characteristics in order to estimate the probability of an individual belonging to a classified sub-group (39). Through this, high and low QOL groups with similar response patterns to QOL-related items can be classified to identify the characteristics of each group and the differences between the 2 groups. Moreover, latent profile classification that accounts for individual characteristics was based on objective statistics, which eliminated the subjectivity of the researchers (40).

Nonetheless, the present study had some limitations. First, the questionnaire was based on a self-report format, and as a result there were many missing values. Consequently, the number of participants included in the final data analysis was lower than the number of participants who took part in the initial survey. The InSCI survey consists of a total of 125 items, which requires an average of 40 min or more to read each item slowly and provide the response. Accordingly, distributing the questionnaire and asking the respondents to self-report the responses may have increased the percentage of items with missing responses. In a future second round InSCI survey, it will be necessary to provide additional explanation (via the form or in person) to reduce the tendency to have missing values.

Second, there were only 2 participating centres: the National Rehabilitation Center and the Korea Spinal Cord Injury Association. This may restrict the representativeness of the sample. The second InSCI survey needs sampling with consideration of institutional characteristics and regional distribution.

Third, while the InSCI survey enables international comparisons, the availability of raw data from other participating countries was limited. This restricted the ability to perform direct statistical comparisons or conduct in-depth cross-national analyses within this study.

### Conclusion

The present study used latent profile files of Koreans with SCI for more objective identification of the factors that influence QOL. To enhance the QOL of community-dwelling Koreans with SCI, providing medical care for bowel dysfunction or spasticity, providing vocational rehabilitation services to facilitate return to work, improving negative perceptions of people with disabilities, and implementing policies to guarantee an income for people with disabilities are needed.

## References

[1] World Health Organization. WHOQOL user manual: Programme on mental health. WHO; 1998. https://www.who.int/tools/whoqol

[2] Nandoe Tewarie RD, Hurtado A, Bartels RH, Grotenhuis JA, Oudega M. A clinical perspective of spinal cord injury. NeuroRehabilitation 2010; 27: 129–139. 10.3233/NRE-2010-0589 20871142

[3] Jensen MP, Truitt AR, Schomer KG, Yorkston KM, Baylor C, Molton IR. Frequency and age effects of secondary health conditions in individuals with spinal cord injury: a scoping review. Spinal Cord 2013; 51: 882–892. 10.1038/sc.2013.112 24126851

[4] Halvorsen A, Pape K, Post MWM, Biering-Sørensen F, Mikalsen S, Hansen AN, et al. Participation and quality of life in persons living with spinal cord injury in Norway. J Rehabil Med 2021; 53: jrm00217. 10.2340/16501977-2858 34232321 PMC8638721

[5] World Health Organization. International perspectives on spinal cord injury; 2013 [cited 2020 Jan 7]. Available from: https://www.who.int/publications/i/item/international-perspectives-on-spinal-cord-injury

[6] Ullrich PM, Spungen AM, Atkinson D, Bombardier CH, Chen Y, Erosa NA, et al. Activity and participation after spinal cord injury: state-of-the-art report. J Rehabil Res Dev 2012; 49: 155–174. 10.1682/JRRD.2010.06.0108 22492345

[7] Strøm V, Månum G, Arora M, Joseph C, Kyriakides A, Le Fort M, et al. Physical health conditions in persons with spinal cord injury across 21 countries worldwide. J Rehabil Med 2022; 54: jrm00302. 10.2340/jrm.v54.2040 35678293 PMC9272839

[8] Nagoshi N, Kaneko S, Fujiyoshi K, Takemitsu M, Yagi M, Iizuka S, et al. Characteristics of neuropathic pain and its relationship with quality of life in 72 patients with spinal cord injury. Spinal Cord 2016; 54: 656–661. 10.1038/sc.2015.210 26620877

[9] Burke D, Lennon O, Fullen BM. Quality of life after spinal cord injury: the impact of pain. Eur J Pain 2018; 22: 1662–1672. 10.1002/ejp.1248 29770520

[10] Andresen SR, Biering-Sørensen F, Hagen EM, Nielsen JF, Bach FW, Finnerup NB. Pain, spasticity and quality of life in individuals with traumatic spinal cord injury in Denmark. Spinal Cord 2016; 54: 973–979. 10.1038/sc.2016.46 27067654

[11] Theisen KM, Mann R, Roth JD, Pariser JJ, Stoffel JT, Lenherr SM, et al. Frequency of patient-reported UTIs is associated with poor quality of life after spinal cord injury: a prospective observational study. Spinal Cord 2020; 58: 1274–1281. 10.1038/s41393-020-0481-z 32409777

[12] Putzke JD, Richards JS, Hicken BL, DeVivo MJ. Predictors of life satisfaction: a spinal cord injury cohort study. Arch Phys Med Rehabil 2002; 83: 555–561. 10.1053/apmr.2002.31173 11932861

[13] Tate DG, Kalpakjian CZ, Forchheimer MB. Quality of life issues in individuals with spinal cord injury. Arch Phys Med Rehabil 2002; 83: S18–S25. 10.1053/apmr.2002.36835 12474168

[14] Post MW, Reinhardt JD, Avellanet M, Escorpizo R, Engkasan JP, Schwegler U, et al. Employment among people with spinal cord injury in 22 countries across the world: results from the international spinal cord injury community survey. Arch Phys Med Rehabil 2020; 101: 2157–2166. 10.1016/j.apmr.2020.05.027 32673653

[15] Reinhardt JD, Middleton J, Bökel A, Kovindha A, Kyriakides A, Hajjioui A, et al. Environmental barriers experienced by people with spinal cord injury across 22 countries: results from a cross-sectional survey. Arch Phys Med Rehabil 2020; 101: 2144–2156. 10.1016/j.apmr.2020.04.027 32502565

[16] Gross-Hemmi MH, Post MW, Ehrmann C, Fekete C, Hasnan N, Middleton JW, et al. Study protocol of the International Spinal Cord Injury (InSCI) community survey. Am J Phys Med Rehabil 2017; 96: S23–S34. 10.1097/PHM.0000000000000647 28059876

[17] Barzallo DP, Gross-Hemmi M, Bickenbach J, Juocevičius A, Popa D, Wahyuni LK, et al. Quality of life and the health system: a 22-country comparison of the situation of people with spinal cord injury. Arch Phys Med Rehabil 2020; 101: 2167–2176. 10.1016/j.apmr.2020.04.030 32533934

[18] Han ZA, Lee BS, Kim W, Lee SJ, Im HJ, Kim C, et al. People with spinal cord injury in Korea. Am J Phys Med Rehabil 2017; 96: S83–S85. 28059886 10.1097/PHM.0000000000000593

[19] Barzallo DP, Gross-Hemmi MH. The cross-cultural societal response to SCI: health and related systems. Am J Phys Med Rehabil 2017; 96: S41–S54. 10.1097/PHM.0000000000000664 28059878

[20] Shin JC, Goo HR, Yu SJ, Kim DH, Yoon SY. Depression and quality of life in patients within the first 6 months after the spinal cord injury. Ann Rehabil Med 2012; 36: 119–125. 10.5535/arm.2012.36.1.119 22506244 PMC3309324

[21] Lee JS, Kim SW, Jee SH, Kim JC, Choi JB, Cho SY, et al. Factors affecting quality of life among spinal cord injury patients in Korea. Int Neurourol J 2016; 20: 316–320. 10.5213/inj.1630540.270 28043106 PMC5209570

[22] Lanza ST, Cooper BR. Latent class analysis for developmental research. Child Dev Perspect 2016; 10: 59–64. 10.1111/cdep.12163 31844424 PMC6914261

[23] WHOQOL Group. Development of the World Health Organization WHOQOL-BREF quality of life assessment. Psychol Med 1998; 28: 551–558. 10.1017/S0033291798006667 9626712

[24] Asparouhov T, Muthen B. Auxiliary variables in mixture modeling: three-step approaches using Mplus. Struct Equ Modeling 2014; 21: 329–341. 10.1080/10705511.2014.915181

[25] Nylund KL, Asparouhov T, Muthen BO. Deciding on the number of classes in latent class analysis and growth mixture modeling: a Monte Carlo simulation study. Struct Equ Modeling 2007; 14: 535–569. 10.1080/10705510701575396

[26] Muthén B. Latent variable analysis: growth mixture modeling and related techniques for longitudinal data. In: Kaplan D, editor. The SAGE handbook of quantitative methodology for the social sciences. Thousand Oaks, CA: Sage; 2004, 345–368. 10.4135/9781412986311.n19

[27] Curran PJ, West SG, Finch JF. The robustness of test statistics to nonnormality and specification error in confirmatory factor analysis. Psychol Methods 1996; 1: 16–29. 10.1037/1082-989X.1.1.16

[28] Adriaansen JJ, Ruijs LE, van Koppenhagen CF, van Asbeck FW, Snoek GJ, van Kuppevelt D, et al. Secondary health conditions and quality of life in persons living with spinal cord injury for at least ten years. J Rehabil Med 2016; 48: 853–860. 10.2340/16501977-2166 27834436

[29] Rivers CS, Fallah N, Noonan VK, Whitehurst DG, Schwartz CE, Finkelstein JA, et al. Health conditions: effect on function, health-related quality of life, and life satisfaction after traumatic spinal cord injury. An observational registry cohort study. Arch Phys Med Rehabil 2018; 99: 443–451. 10.1016/j.apmr.2017.06.012 28732686

[30] Bloemen-Vrencken JH, Post MW, Hendriks JM, De Reus EC, De Witte LP. Health problems of persons with spinal cord injury living in the Netherlands. Disabil Rehabil 2005; 27: 1381–1390. 10.1080/09638280500164685 16321920

[31] Kim SH, Kim O, Bae YH, Choi DI, Heo JE, Song WK, et al. Caregiver burden according to ageing and type of care activity in caregivers of individuals with spinal cord injury. Spinal Cord Ser Cases 2023; 9: 16. 10.1038/s41394-023-00570-w PMC10113187 37072384

[32] Kent ML, Dorstyn DS. Psychological variables associated with employment following spinal cord injury: a meta-analysis. Spinal Cord 2014; 52: 722–728. 10.1038/sc.2014.92 25091108

[33] Ottomanelli L, Barnett S, Goetz L, Toscano R. Vocational rehabilitation in spinal cord injury: what vocational service activities are associated with employment program outcome? Top Spinal Cord Inj Rehabil 2015; 21: 31–39. 10.1310/sci2101-31 25762858 PMC4349173

[34] Krause JS, Terza JV, Dismuke CE. Factors associated with labor force participation after spinal cord injury. J Vocat Rehabil 2010; 33: 89–99. 10.3233/JVR-2010-0518

[35] Reinhardt JD, Mansmann U, Fellinghauer BA, Strobl R, Grill E, von Elm E, et al. Functioning and disability in people living with spinal cord injury in high- and low-resourced countries: a comparative analysis of 14 countries. Int J Public Health 2011; 56: 341–352. 10.1007/s00038-010-0222-8 21165668

[36] Filipcic T, Sember V, Pajek M, Jerman J. Quality of life and physical activity of persons with spinal cord injury. Int J Environ Res Public Health 2021; 18: 9148. 10.3390/ijerph18179148 34501739 PMC8430911

[37] Lee BS, Kim O, Ham D. Epidemiological changes in traumatic spinal cord injuries for the last 30 years (1990–2019) in South Korea. Spinal Cord 2022; 60: 612–617. 10.1038/s41393-021-00694-6 34465888

[38] Geyh S, Peter C, Müller R, Bickenbach JE, Kostanjsek N, Üstün BT, et al. The personal factors of the International Classification of Functioning, Disability and Health in the literature: a systematic review and content analysis. Disabil Rehabil 2011; 33: 1089–1102. 10.3109/09638288.2010.523104 20925452

[39] Lanza ST, Rhoades BL. Latent class analysis: an alternative perspective on subgroup analysis in prevention and treatment. Prev Sci 2013; 14: 157–168. 10.1007/s11121-011-0201-1 21318625 PMC3173585

[40] Giang MT, Graham S. Using latent class analysis to identify aggressors and victims of peer harassment. Aggress Behav 2008; 34: 203–213. 10.1002/ab.20233 17828767

